# Bioinspired bioadhesion: translating nature’s adhesive strategies into regenerative medicine

**DOI:** 10.1007/s44258-026-00080-y

**Published:** 2026-04-01

**Authors:** Sushila Maharjan, Jacqueline Jialu He, David Hyram Hernández Medina, Bibhor Singh, Fabiola Chapa, Tsandni Wasram Jetha-Jamal, Yu Shrike Zhang

**Affiliations:** 1 Division of Engineering of Medicine, Department of Medicine, Brigham and Women’s Hospital, Harvard Medical School, Cambridge, MA 02139 USA; 2 Belmont Middle School, Belmont, MA 02478 USA; 3 Harvard Stem Cell Institute, Harvard University, Cambridge, MA 02138 USA; 4 Broad Institute of MIT and Harvard, Cambridge, MA 02142 USA

**Keywords:** Bioadhesive, Bioinspired, Biomaterials, Hydrogels, Regenerative medicine

## Abstract

In nature, many organisms, such as mussels, geckos, tree frogs, octopuses, and salamanders, have evolved remarkable bioadhesion strategies, that enable them to attach to wet environments, climb vertical or inverted surfaces, and capture preys. These strategies rely on chemical interactions mediated by secreted bioadhesives as well as physical forces, including but not limited to friction, van der Waals interactions, capillary forces, and vacuum suction, arising from specialized micro- and nanostructures. Chemical bioadhesives, composed of proteins, polysaccharides, or other macromolecules, facilitate strong, reversible or irreversible adhesion to wet or dynamic surfaces, as exemplified by mussel byssal threads and tree frog toe pad mucus. These adhesives act through mechanisms such as covalent bonding, metal coordination, hydrogen bonding, and electrostatic interactions. This review outlines recent advances in both chemical and physical bioadhesion strategies. We examine the adhesion principles used by mussels, geckos, tree frogs, octopuses, and other organisms that secrete adhesive chemicals, emphasizing the roles of micro- and nanostructures, interfacial forces, and soft contact mechanics. We also present design strategies for creating artificial adhesives inspired by these biological systems and describe their applications in regenerative medicine. Finally, we discuss current challenges and future directions in bioinspired and chemically based adhesion.

**Graphical Abstract:**

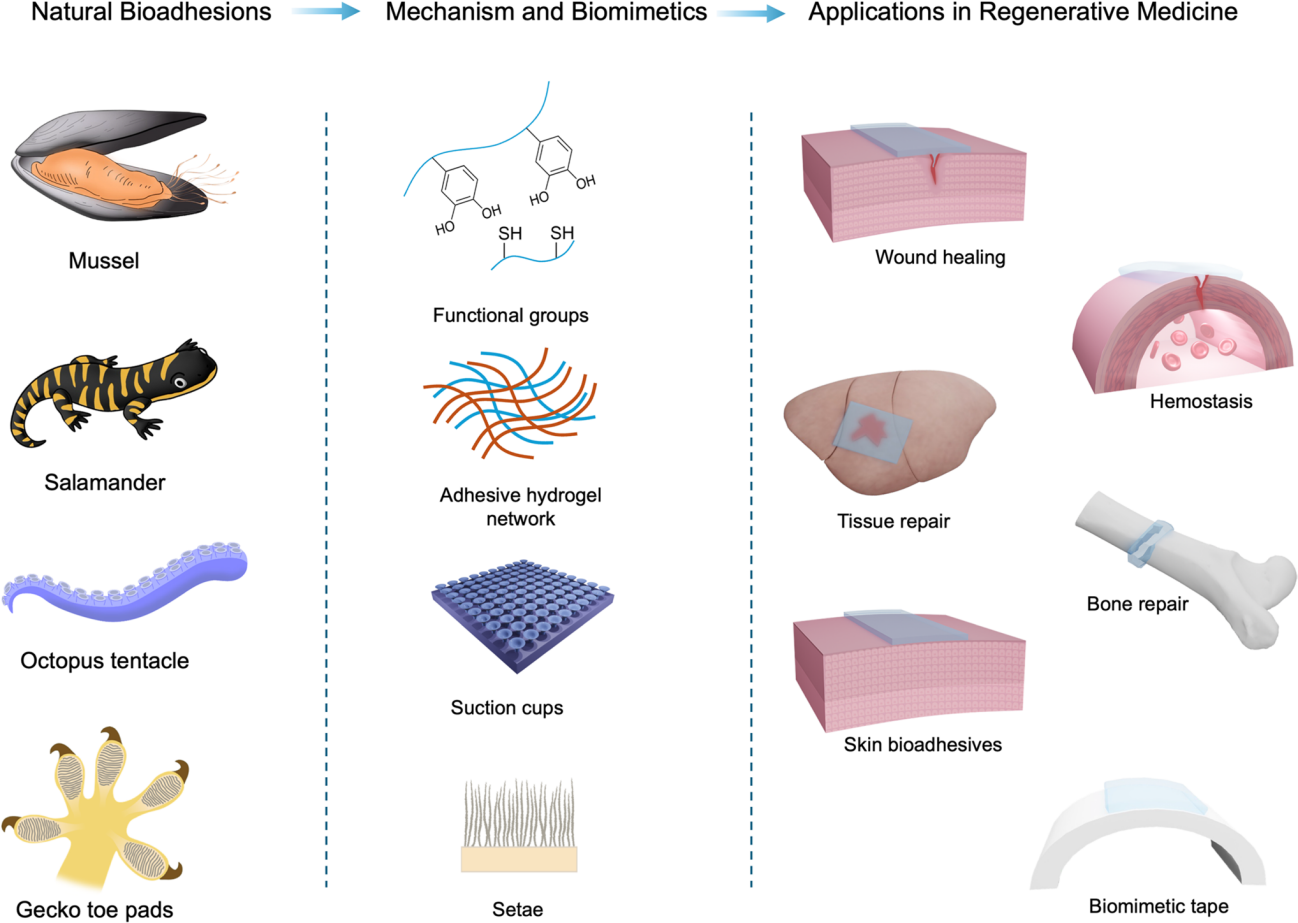

## Highlights

- Diverse natural bioadhesives and bioadhesion strategies in aquatic and terrestrial environments.
- Physicochemical bioadhesion mechanisms in selected aquatic and terrestrial organisms.
- Bioinspired synthetic adhesives, and micro-/nanoscale bioadhesive systems for regenerative medicine applications.

## Introduction

Adhesion in biological systems is fundamental to survival, growth, and healing. Nature has evolved a multitude of adhesion mechanisms across diverse species to address a wide spectrum of environmental and biological challenges [1–4]. These natural adhesion mechanisms primarily arise from specialized secretions with distinct and often complex chemical compositions, and/or the presence of intricate micro- and nanostructures on the surfaces of body parts. These mechanisms enable adhesion to a variety of surfaces, including wet, dry, dynamic, submerged, and biofouling-prone environments, and support essential biological processes such as wound healing, tissue regeneration, structural stability, as well as resistance to mechanical and environmental stresses [5–7]. Such systems are vital for various biological functions, including movement, reproduction, survival, and interactions within ecosystems [4, 5, 8–10].

Aquatic organisms have evolved robust bioadhesive systems to function in challenging underwater environments, where factors such as moisture, salinity, and biofouling present significant obstacles [7, 11]. These methods rely on wet adhesion mechanisms that are specially adapted to function in submerged, often turbulent, and high-humidity conditions. Marine species such as mussels, barnacles, and sea stars secrete complex protein-based glues or composite materials that can displace water, form strong cohesive bonds, and adhere robustly to wet and irregular surfaces such as rocks, shells, and even metal [9, 12]. These bioadhesives typically involve oxidative crosslinking, metal ion coordination, and hydrophobic interactions to ensure strong, permanent bonding and mechanical cohesion in wet environments [13–16]. For example, mussels produce foot proteins rich in 3,4-dihydroxyphenylalanine (DOPA), which mediate catechol-mediated adhesion through both covalent and non-covalent bonding [12, 17, 18]. Similarly, barnacles secrete a cement-like substance composed of multiple cement proteins that polymerize underwater [19, 20].

In addition to bioadhesive secretions, some marine species have evolved mechanical adhesion strategies to interact with submerged surfaces. For example, octopuses utilize highly dynamic, soft suckers composed of muscular hydrostats, which allow them to conform to and grip a wide variety of irregular or smooth underwater surfaces [21–23]. These suckers create strong suction by actively manipulating internal pressure and surface contact area, enabling precise and reversible attachment even in turbulent or cluttered environments [22, 24].

While aquatic biological adhesives have received significant attention due to their relevance to the wet and dynamic conditions encountered within the human body, terrestrial organisms have also evolved a wide range of adhesion strategies to cope with complex, dry, wet, or heterogeneous environments [23]. Terrestrial bioadhesion integrates biophysical principles, such as capillarity, surface tension, and mechanical interlocking, with biochemical strategies involving glycoproteins, lipids, and surfactants [25, 26]. These systems utilize either dry adhesion, as seen in geckos with their hierarchically structured toe pads relying on van der Waals forces [27, 28]; or wet adhesion, found in species like tree frogs where they use their mucus-rich toe pads for adhesion on moist surfaces [26, 29]. Moreover, salamanders such as giant salamander produce adhesive secretions from specialized skin glands as an anti-predator defense or for wound sealing [30–32].

While natural bioadhesives produced by both aquatic and terrestrial organisms offer nontoxic, biodegradable, and robust alternatives to conventional synthetic medical adhesives [33–35], their structural and chemical diversity, shaped over millions of years of evolution and natural selection, provide powerful biological blueprints for the design of biomimetic adhesives [4, 36]. Natural bioadhesive secretions are increasingly studied not only to understand their underlying chemistry but also to inspire the development of biomimetic adhesives for biomedical and industrial applications [2, 3, 16, 34, 37]. Bioinspired adhesives derived from aquatic systems are especially promising for internal medical applications, particularly for use in internal wound closure, tissue repair, and implant fixation [3]. Unlike traditional sutures or staples, bioinspired adhesives can offer minimally invasive, flexible, and biodegradable solutions that conform to wet tissues, reduce inflammation, and promote healing [38, 39]. Ongoing research into the molecular mechanisms of marine bioadhesion is driving innovations in surgical glues, drug delivery systems, and regenerative scaffolds designed to perform in the dynamic and hydrated environment of the human body [40, 41]. For instance, mussel-inspired adhesives are being developed for wound closure, cartilage repair, and localized drug delivery [2, 42].

Similarly, bioadhesion strategies that integrate mechanical mechanisms with material properties enable firm adhesion in challenging environments, such as underwater. As discussed, barnacles secrete permanent protein-based glues that adhere strongly to submerged surfaces, while octopuses employ suction and microstructural adaptations for reversible attachment. These principles have inspired technologies including soft robotics, underwater gripping devices, and medical suction systems, among others [2, 4, 43–45]. Similarly, the dry, non-permanent adhesion mechanics of gecko feet have led to the development of synthetic adhesives with controllable adhesion and easy detachment, and climbing robots, with applications ranging from minimally invasive surgery to space exploration and prosthetics [46–49]. Likewise, insights from other terrestrial systems, such as the wet adhesive secretions of tree frogs, further guide the design of reusable and environmentally responsive materials, which are particularly valuable for smart scaffolds, temporary implants, and biointerfaces [40, 50, 51].

This review accordingly highlights the remarkable diversity of natural bioadhesives and bioadhesion strategies that have evolved across aquatic and terrestrial environments. We explore how organisms have evolved specialized adhesion systems adapted to the specific demands of their environments, from the permanent underwater bioadhesives produced by mussels and barnacles to the reversible suction-based adhesion used by octopuses, all of which are uniquely adapted to function effectively in wet conditions. In (semi-)terrestrial species, we discuss the bioadhesive skin secretions of salamanders and mucin secretions from tree frog toe pads. In addition, we also present mechanical adhesion strategies observed in species such as geckos, which achieve dry, non-permanent adhesion through specialized structural adaptations rather than the use of secreted substances. By analyzing the chemical, structural, and mechanical principles underlying these systems, this review emphasizes how organisms have evolved a range of solutions to function effectively in extreme and dynamic environments. Beyond deepening our understanding of biological adhesion, we also highlight how these natural strategies provide valuable design principles for the development of next-generation biomimetic adhesives, with promising applications in regenerative medicine and beyond.

## Natural bioadhesives

**Fig. 1:**
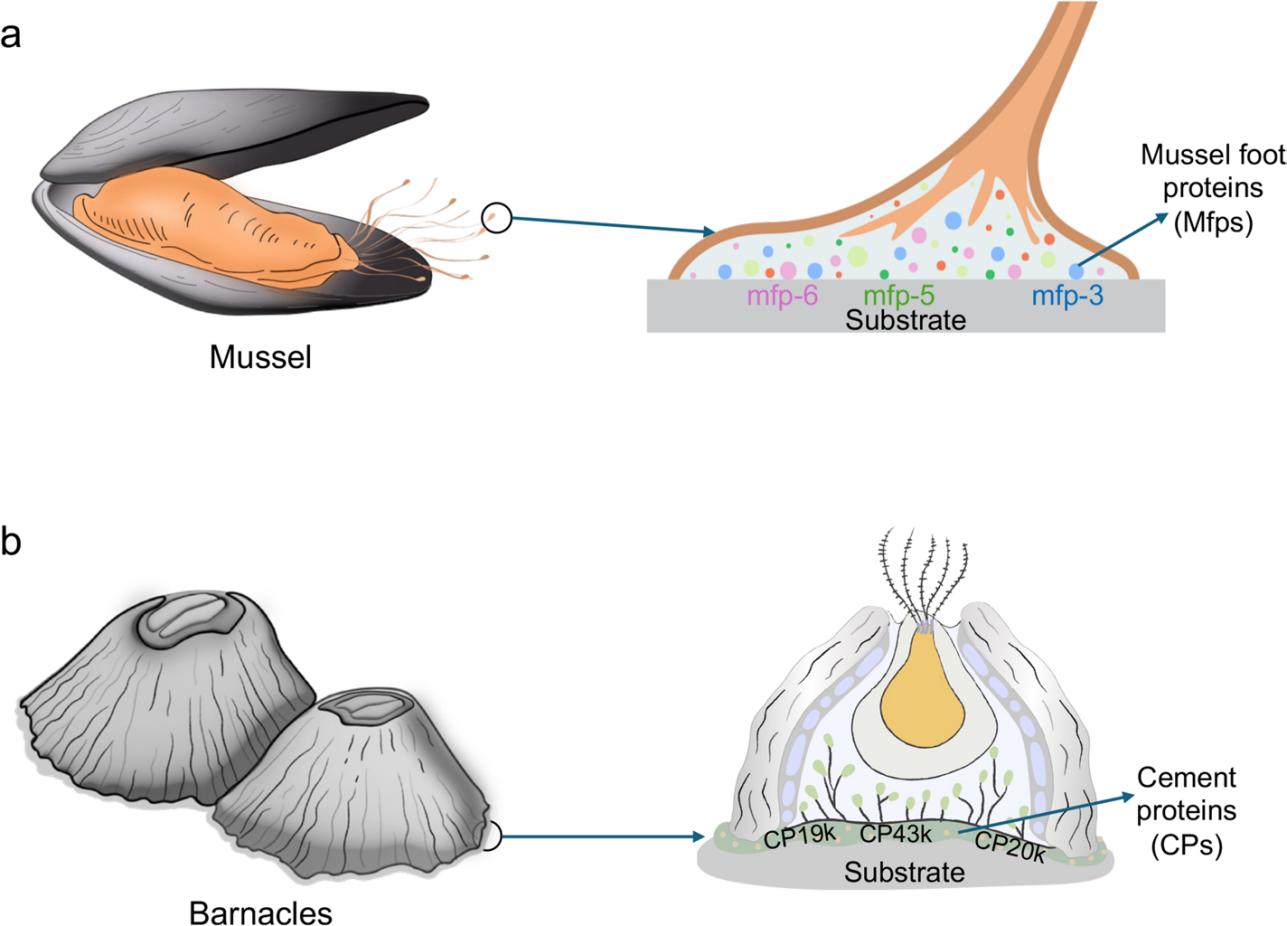
Natural chemical bioadhesives. Nature has evolved a wide range of bioadhesives, sticky substances secreted by organisms to enable stable attachment to different surfaces. **a** Mussels produce foot proteins containing catechol groups that form covalent and non-covalent bonds with surfaces. **b** Barnacles secrete cement proteins to form permanent bonding to surfaces in wet environments

Bioadhesion via secreted chemical bioadhesives refers to attachment achieved through the release of specialized sticky secretions by organisms. These bioadhesives are typically composed of proteins, polysaccharides, or other macromolecules capable of forming cohesive networks and establishing strong interfacial interactions with a wide range of substrates [52, 53]. While bioadhesion mechanisms are diverse and complex, depending on the ecological and functional demands of each organism, secreted bioadhesives can mediate either irreversible and permanent adhesion or reversible and temporary adhesion [7, 35, 54]. Irreversible and permanent adhesion is mediated by bioadhesives that form strong bonds, often through chemical crosslinking of their molecular components. These crosslinks stabilize the adhesive matrix, making it resistant to degradation and detachment even under challenging environmental conditions such as immersion in water, exposure to varying pH or salinity, and mechanical stress from turbulent flows [55, 56]. A classic example is the marine mussel, which secretes adhesive proteins containing catechol groups (*e.g.*, DOPA) that form covalent and non-covalent bonds with surfaces (Fig. 1a) [57–59]. Likewise, barnacles produce cement proteins that polymerize into a densely crosslinked network, providing exceptionally strong and long-lasting adhesion (Fig. 1b) [19, 37]. Such permanent adhesions are essential for sessile organisms that depend on stable, long-term attachment for feeding, reproduction, and survival in dynamic underwater habitats [15].

**Fig. 2:**
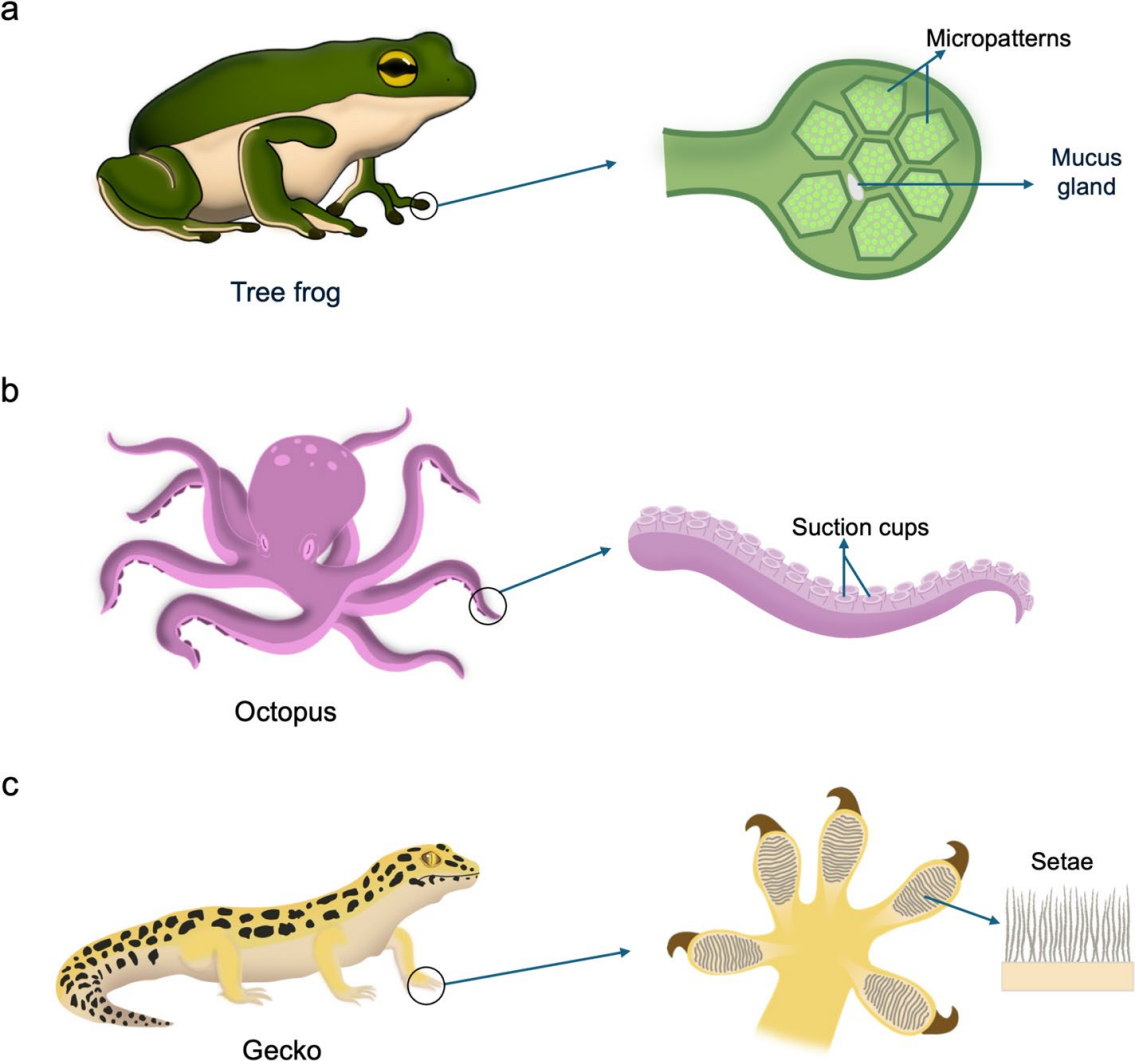
Natural structural bioadhesion systems. The intricate micro- and nanostructural adaptations in some organisms provide powerful and reversible adhesion in various environments. **a** Tree frogs use their mucus-rich toe pads with micropatterns for adhesion on wet surfaces. **b** Octopi use highly dynamic, soft suckers to attach to variety of underwater surfaces. **c** Geckos utilize their hierarchically structured toe pads for adhesion on dry surfaces

On the other hand, reversible and temporary adhesions are mediated by bioadhesives that form weaker interactions with surfaces [15]. Unlike permanent adhesives, these temporary adhesives often depend on hydrogen bonding, electrostatic interactions, or reversible coordination bonds that can be broken and re-formed with minimal energy [34, 60]. These adhesives often remain hydrated and pliable, enabling the organism to release and reattach multiple times without losing effectiveness. This strategy provides flexibility in dynamic environments or behaviors that require frequent movements. For example, slugs and snails secrete a thin layer of pedal mucus rich in glycoproteins and other macromolecules, which mediates reversible adhesion while simultaneously providing lubrication [60, 61]. This dual function enables them to glide smoothly over surfaces, including vertical or inverted planes, and to detach easily whenever needed. Similarly, amphibians such as tree frogs secrete mucus on the bottom of their micropatterned toe pads, enabling them to adhere to surfaces through capillary and hydrodynamic forces (*i.e.*, wet adhesion) (Fig. 2a) [62, 63]. Similarly, salamanders produce sticky skin secretions that facilitate reversible adhesion for climbing and grasping surfaces as well as provide protection against predators [32, 64]. Thus, these secretions enable secure attachment while remaining reversible, allowing rapid detachment for movement, predation, or escape [30, 65]. Together, these examples illustrate how reversible chemical bioadhesion provides both stability and mobility in terrestrial species. Furthermore, micro- and nanoscale structural adaptations, like the suction cups of octopus (Fig. 2b) or setae on gecko feet (Fig. 2c), facilitate non-permanent adhesion in both wet and dry environments by maximizing contact area [66], dissipating energy under stress at the micro- [67] and nanoscales [68] and enabling controlled detachment [69].

## Mechanisms of bioadhesion

**Fig. 3:**
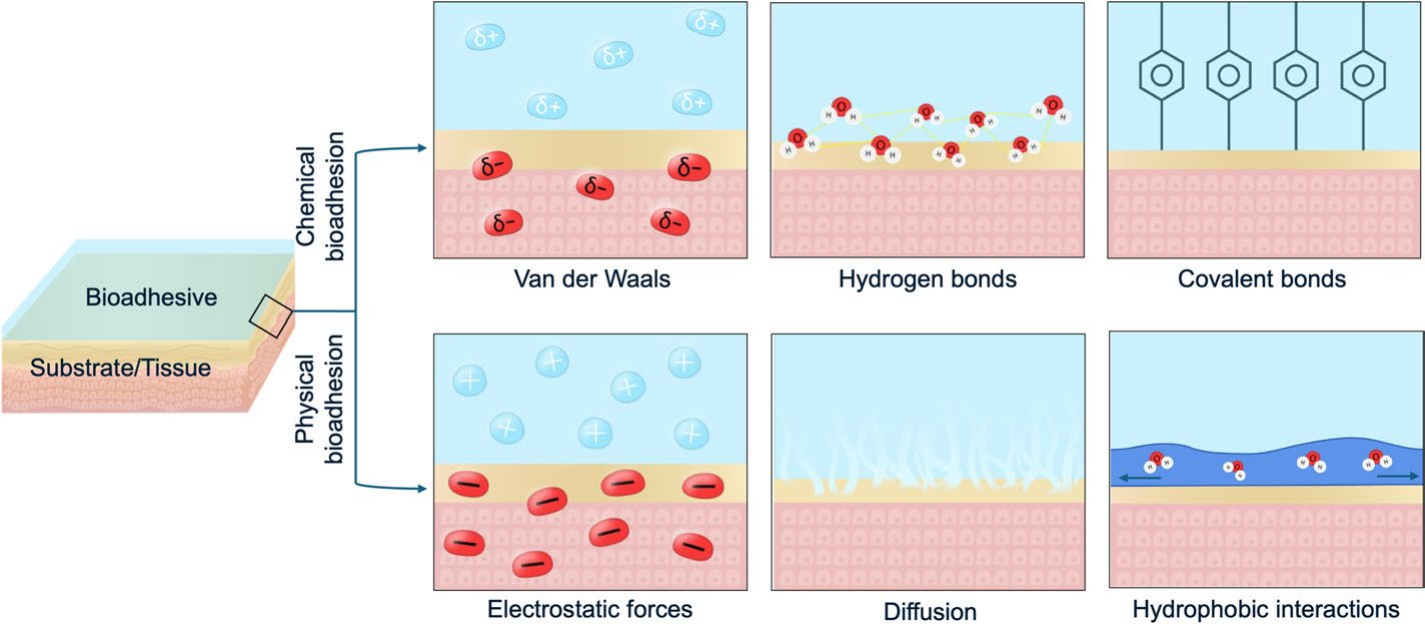
Typical mechanisms of bioadhesion. Schematic representations of typical chemical and physical bioadhesion strategies for bioadhesives

Adhesion in biological systems generally operates through two main mechanisms: wet adhesion and dry adhesion. Chemical principles of bioadhesion rely on molecular interactions and bonding between adhesive secretions and the surface of attachment. These mechanisms are central to wet adhesion, where organisms produce specialized substances, such as mucus or adhesive proteins, that facilitate secure attachment to surfaces. Bioadhesive proteins often contain functional groups, such as amino (–NH_2_), hydroxyl (–OH), sulfhydryl (–SH), and carboxylic acid (–COOH) groups, capable of forming hydrogen bonds and ionic interactions with polar or charged surfaces, ensuring strong yet flexible attachment. In some cases, adhesion is stabilized by covalent bonding, such as the crosslinking of catechol-rich amino acids, which provides strong adhesion to the surface (Fig. 3).

In contrast, dry adhesion does not require secretions but instead often relies on direct physical interactions between surfaces. Physical adhesion forces are highly dependent on factors such as surface roughness and wettability of the substrate, mechanics of force application and material properties of the adhesives [16, 58, 70, 71]. Geckos achieve dry adhesion using arrays of microscopic setae on their toe pads, which maximize contact area and generate van der Waals forces, enabling reversible attachment to a variety of surfaces. Capillary forces, observed in tree frogs, rely on thin mucus films that create surface tension to secure attachment. In certain cases, in addition to adhesive proteins, hydrophobic interactions further enhance adhesion by displacing interfacial water and promoting close contact between adhesive molecules and the substrate. For example, marine mussels use hydrophobic domains in their adhesive proteins to attach firmly to wet rocks, even in turbulent water. Additionally, hydrodynamic forces contribute by generating fluid flow and pressure differences in organisms that secrete mucus onto surfaces. Snails, for instance, use mucus-mediated hydrodynamic forces to maintain strong adhesion while moving across wet or flowing surfaces. Another important mechanism is electrostatic forces, which is based on the attraction of oppositely charged surfaces to support adhesion in certain organisms (Fig. 3).

Therefore, the effectiveness of bioadhesive interfaces sometimes is not provided by chemical interactions alone [72], but can also be enhanced with physical forces and mechanical principles that work synergistically with secreted bioadhesives to allow organisms to attach, detach, and adapt to both wet [73] and dry [74] environments. In this section, the mechanisms of bioadhesion are described by examining the physicochemical processes involved in two aquatic and two terrestrial organisms. In addition, we highlight physical bioadhesion strategies by analyzing two representative processes, one in an aquatic organism and one in a terrestrial organism, emphasizing their mechanisms of action and potential applications.

### Bioadhesion mechanism of mussels

**Fig. 4:**
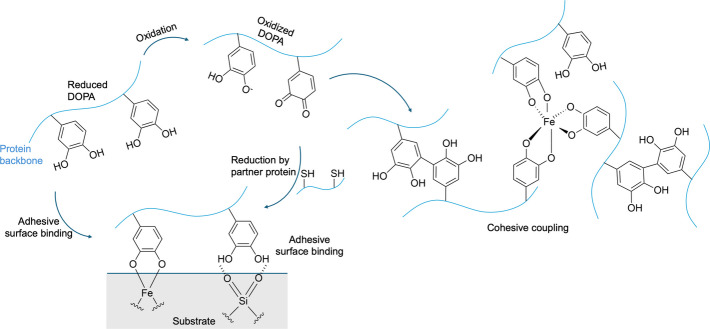
Mechanism of DOPA-mediated bioadhesion in mussels. The reduced DOPA directly form covalent and non-covalent bonds with substrate surfaces to achieve bioadhesion, while cohesion arises through metal ion coordination and oxidative crosslinking. Reproduced with permission from [84]

Mussels are among the best-studied marine organisms that achieve strong adhesion in wet and dynamic environments. Mussels produce fibrous structures termed byssal threads, which terminate in adhesive plaques that anchor them to a wide range organic and inorganic surfaces. These plaques are composed largely of mussel foot proteins (Mfps) whose key component is the catechol-containing DOPA, that enables adhesion by forming hydrogen bonds, metal coordination bonds, and covalent bonds with both the substrate and neighboring proteins [75–77]. More importantly, they have evolved a special mechanism to control the adhesion strength over time [78]. In freshly secreted Mfps, the catechol group of dopa forms noncovalent interactions with substrates, including charge–charge, π–π, cation–π, and hydrophobic interactions as well as metal coordination and hydrogen bonding [3, 57, 76, 79]. Over time, DOPA is oxidized to dopaquinone, which forms hydrogen bonds with unoxidized catechol and amino or carboxyl groups, while its hydrophobic aggregation further enhances cohesion [3, 57]. Finally, dopaquinone undergoes slow covalent crosslinking with primary amines and thiols resulting in permanent adhesion (Fig. 4) [80–83]. This time-dependent adhesion is regulated by the pH of the environment and the enzymatic activity. Remarkably, through the complex chemistry of DOPA, mussels can achieve both strong and rapid adhesion while also regulating the process to avoid premature hardening or adhesion to the wrong surface.

### Bioadhesion mechanism of barnacles

Barnacles are sessile marine crustaceans well-known for their unique and robust adhesive system that enables permanent underwater attachment to a broad range of substrates, including rocks and ship hulls. Their adhesion is mediated by a complex, proteinaceous cement composed of multiple cement proteins (CPs), which act synergistically to ensure strong bonding and mechanical cohesion in wet environments [56, 85]. Unlike the catechol-mediated adhesion found in mussels, barnacle adhesion is DOPA-independent. Instead, CPs are rich in phosphorylated residues (*e.g.*, serine, threonine) and cysteines, supporting both electrostatic and disulfide-mediated crosslinking [86, 87]. Thus, they use thiol-rich and phosphorylated proteins to mediate adhesion, showing an alternative evolutionary strategy for underwater adhesion.

To date, over a dozen CPs have been identified in barnacles, particularly in species such as *Amphibalanus amphitrite*, of which at least six types of CPs have been found to play distinct roles in cohesion and surface binding [56, 88, 89]. CPs are generally categorized based on their molecular weight and function. While barnacle cement consists primarily of CPs (> 90%), smaller amounts of carbohydrates, inorganic compounds, and lipids are present as well. The robust adhesive strength is largely attributed to synergistic interactions between CPs and lipids [90, 91].

The barnacle cement operates through two main phases. Initially some CPs serve as primers, facilitating bonding to the substrate, while other CPs polymerize to form the bulk of the adhesive, providing mechanical strength and cohesion [90, 92]. CP19k is believed to play a critical role in the initial surface adhesion phase. It is enriched in hydrophilic and charged residues, which allow it to interact with a variety of substrates, including metals, polymers, and natural surfaces [93]. CP20k is thought to assist in substrate recognition and interface compatibility and modulate the interfacial properties of the substrate, improving cement spreadability and wetting [88]. CP43k and CP52k are involved in forming the structural matrix of the adhesive [94, 95]. These CPs are rich in cysteine, serine, and glycine, suggesting potential for disulfide bonding and *β*-sheet formation, which enhance the stability of the adhesive network. The CPs contribute significantly to the cohesive strength of the cement, enabling mechanical durability under dynamic marine conditions [94]. CP68k is thought to play a structural role by acting as a molecular crosslinker, stabilizing interactions between other CPs [96]. High molecular weight CPs, such as CP100k and CP150k, are believed to stabilize barnacle adhesive over time and improve its toughness [89]. The repetitive motifs they contain favor intermolecular interactions, similarly to those in collagen or silk, enabling network entanglement and mechanical reinforcement. Recent studies have revealed that some CPs (*e.g.*, CP52k, CP100k) may form amyloid-like nanofibrils, contributing to the insolubility, mechanical strength, and resistance to proteolysis characteristic of barnacle cement [87]. Its insolubility, resistance to degradation, and strong bonding under wet conditions make it especially effective for long-term underwater adhesion.

Understanding barnacle bioadhesion is of significant interest in marine antifouling technologies, surgical adhesives, and the development of synthetic underwater glues. The unique mechanisms offer insights into designing strong, water-resistant adhesives without relying on the DOPA chemistry.

### Bioadhesion mechanism of tree frog

**Fig. 5:**
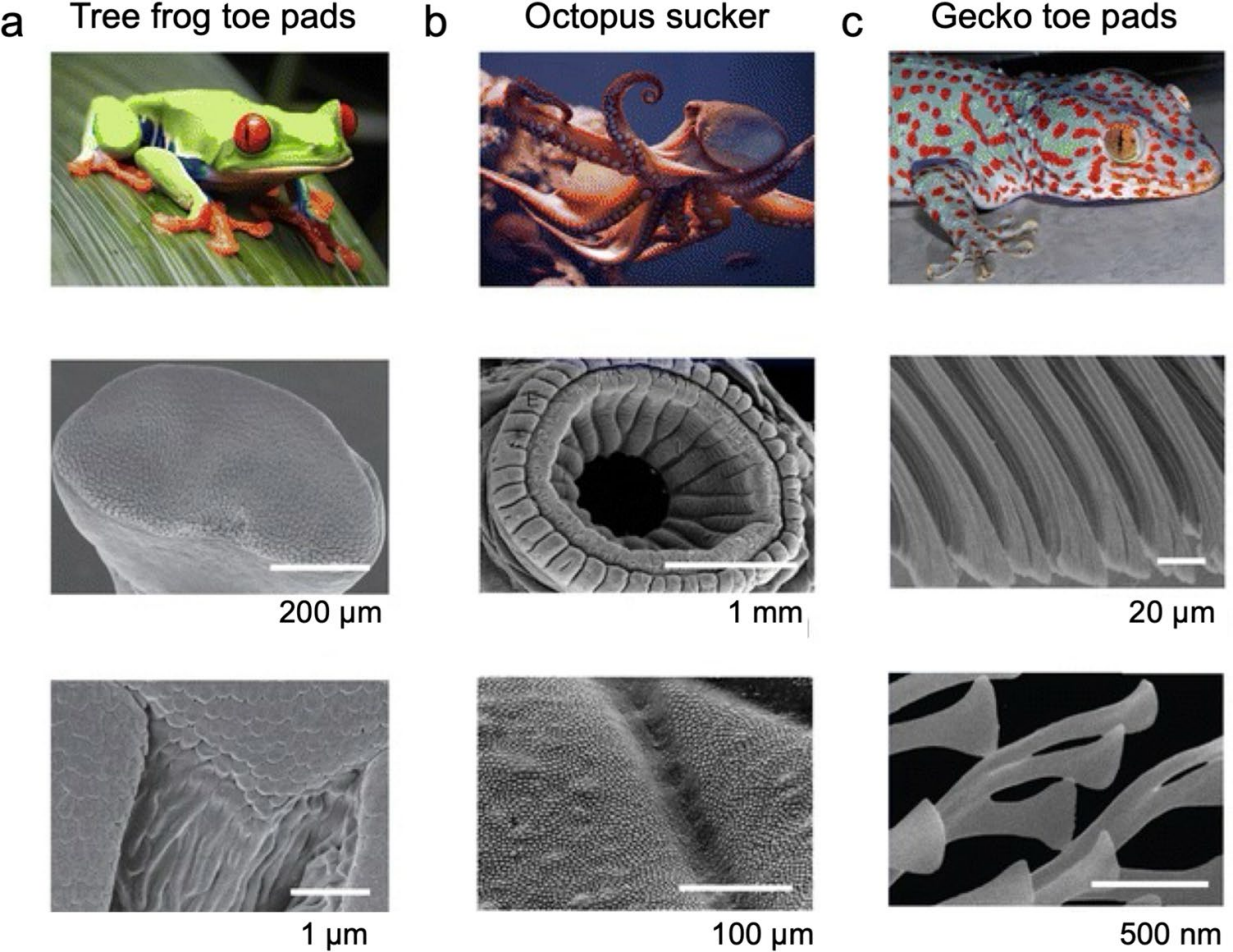
Bioadhesion strategies exhibiting hierarchical multiscale architectures of organisms. **a** Microstructure of a tree frog’s toe pad with micropatterned epithelia. Reproduced with permission from [97]. **b** Microstructures of octopus’s sucker rim surface showing numerous projections or denticles. Reproduced with permission from [98]. **c** Gecko toe pads consist of hundreds of thousands of setae and each seta contains hundreds of spatulae. Reproduced with permission from [99]

Tree frogs achieve adhesion primarily through a combination of specialized toe pad structures and mucin-rich mucus secretion [62, 63]. Their toe pads are composed of hexagonal epithelial cells separated by channels that facilitate mucus distribution and drainage, maximizing contact with smooth or irregular surfaces (Fig. 5a) [62]. The adhesive mucus is a viscoelastic hydrogel primarily made of mucins, heavily glycosylated glycoproteins, along with water, salts, and minor lipids [62]. These mucins form flexible networks that trap water, maintain hydration, and create a thin fluid film that generates capillary and viscous forces, providing strong yet reversible adhesion, even on smooth or wet substrates [63]. In addition, the soft, deformable pad surface maximizes contact area, while shear forces applied during movement increase friction and allow controlled detachment [29]. This integration of pad microstructure and mucin-based mucus enables tree frogs to grip effectively to both dry and wet surfaces while maintaining the ability to release their grip when needed.

### Bioadhesion mechanism of octopus

Octopus adhesion is achieved through a combination of muscular suction, surface microstructures, and mucus-mediated bioadhesion [98]. Each octopus arm is covered with hundreds of suckers, which can actively create negative pressure to attach to surfaces (Fig. 5b). The suction is enhanced by the flexible, muscular architecture of the sucker, allowing it to conform to both smooth and rough surfaces, forming an airtight seal [21, 98]. In addition to suction, octopuses secrete a mucus-like bioadhesive composed primarily of glycoproteins, polysaccharides, and minor lipids, forming a viscoelastic hydrogel [100]. The glycoproteins in this secretion contain repetitive motifs that promote intermolecular interactions, contributing to cohesion within the adhesive layer, while the polysaccharides enhance hydration and viscoelasticity, allowing the adhesive to maintain grip in wet or dynamic environments [101]. This combination of suction and mucus-mediated adhesion provides strong yet reversible attachment, enabling the octopus to detach quickly by relaxing the sucker muscles and disrupting the mucus network [22, 24]. The synergy between mechanical and chemical adhesion allows octopuses to attach to diverse substrates underwater, while the mucus layer protects the soft tissues of the suckers from damage and prevents surface erosion.

### Bioadhesion mechanism of gecko

Gecko bioadhesion is achieved through a highly specialized hierarchical toe pad structure [6]. Their toe pads are covered with millions of microscopic hair-like setae, each branching into hundreds of nanoscale spatulae, which are flattened, triangular tips that can conform to surface irregularities (Fig. 5c). This hierarchy, from toes to setae to spatulae, maximizes the contact area at the molecular scale, enabling adhesion primarily through dry, non-chemical van der Waals interactions [27]. Although van der Waals forces are individually weak, the enormous number of spatulae collectively generates significant adhesion, enough to support multiple times the gecko’s body weight, yet remains reversible for rapid detachment and reattachment. Geckos control adhesion mechanically by adjusting the angle and shear of the setae [28]. Pulling the setae at a low angle increases contact and adhesion, while changing the angle or peeling the toe tips reduces contact, allowing detachment [102]. This directional control enables selective attachment and release on different toes while maintaining grip during climbing [103].

The flexible spatulae and hierarchical design allow geckos to adhere to a wide variety of surfaces, smooth, rough, dry, or slightly wet, without relying on chemical secretions and distinguishing their dry adhesion strategy from mucus-based bioadhesion in organisms like tree frogs or octopi. Overall, gecko adhesion is a purely physical, hierarchical system where van der Waals forces and directional control properties work together to achieve strong, reversible, and repeatable adhesion across diverse environments.

### Bioadhesion mechanism of salamanders

Salamanders exhibit reversible bioadhesion primarily through mucus secretion combined with specialized toe pad structures [32]. Their toe pads are soft, deformable, and often slightly flattened to increase contact area with surfaces. The adhesive mucus secreted by salamander skin is a glycoprotein-rich hydrogel, similar to that of tree frogs, containing mucins and other macromolecules that form a viscoelastic layer between the pad and the substrate [30]. This layer generates adhesion through capillary forces and viscous interactions, allowing the salamander to grip wet or uneven surfaces effectively. The viscoelastic nature of the mucus also enables reversible attachment, the salamander can release its grip rapidly by peeling or sliding its toes, breaking the capillary bridges without damaging its pads. Additionally, the mucus provides lubrication, which prevents tissue damage and aids in smooth locomotion, while its hydration helps maintain adhesion in humid or aquatic environments. The combination of deformable toe pads and mucin-rich mucus allows salamanders to attach to diverse substrates, balancing strong adhesion with easy detachment, which is critical for climbing, walking on vertical surfaces, and navigating complex environments.

In addition to bioadhesion strategies, salamanders have evolved multiple defensive strategies [104], including tail autotomy, protective camouflage, and toxic or mucous skin secretions, the latter being the most effective [104, 105]. North American species such as *Plethodon shermani*, *Ambystoma spp*., and *Bolitoglossa spp*. secrete adhesive mucus as a defense mechanism [106–109]. In *Plethodon shermani*, two types of cutaneous exocrine glands produce mucus, widely distributed mucous glands secrete acidic glycoproteins with a flocculent appearance and localized granular glands in the parotid and tail regions secrete protein-rich, granular mucus containing basic proteins, glycoconjugates (mannose, *α*-L-fucose), and lipids [106, 107]. The secretions are approximately 70% water, with ~ 78% of the dry content comprising proteins responsible for adhesion [30]. Upon exposure to air, the mucus quickly hardens, enhancing adhesion, although the dehydration-dependent adhesive mechanism and its physical properties remain underexplored [108, 110]. This water-dependent hardening also influences potential clinical applications, such as wound fixation, where evaporation rate governs the adhesive performance.

Recently, an adhesive hydrogel derived from the skin secretion of the Chinese giant salamander (*Andrias davidianus*), termed SSAD, has been developed and evaluated for biomedical applications [111, 112]. This hydrogel demonstrates significantly stronger tissue adhesion compared to conventional fibrin glue, likely due to the high content of glycoproteins and viscoelastic macromolecules in the salamander secretion, which promote capillary and cohesive forces at the tissue interface. In addition to superior adhesion, SSAD exhibits enhanced elasticity, allowing it to accommodate dynamic tissue movements without detachment, and excellent biocompatibility, showing minimal cytotoxicity and inflammatory response in both ex vivo and in vivo studies [32, 113]. Unlike synthetic adhesives such as cyanoacrylate glue, which can be brittle or cytotoxic, SSAD forms a flexible, hydrated network that integrates with tissue surfaces and supports reversible, strong adhesion under physiological conditions. These properties suggest that SSAD-based hydrogels could serve as a promising next-generation tissue adhesive for wound closure, hemostasis, and regenerative medicine applications.

## Bioinspired chemical adhesives

Bioinspired chemical adhesives have emerged as a promising class of synthetic biomaterials designed to replicate the remarkable adhesion strategies observed in nature. Many organisms, such as mussels, slugs, and tree frogs, secrete specialized chemicals that enable strong, reversible, or environmentally resilient adhesion under wet or dry challenging environment. Inspired by these natural mechanisms, researchers have developed synthetic adhesives that mimic key chemical functionalities, such as catechols, proteins, glycoproteins, and polysaccharides, to achieve tunable adhesion, biocompatibility, and durability. These bioinspired chemical adhesives utilize mechanisms including hydrogen bonding, covalent crosslinking, and capillary-assisted adhesion to achieve strong and versatile performance on a variety of surfaces. By translating natural adhesion strategies into engineered biomaterials, synthetic bioadhesives offer potential applications in medicine, robotics, and industrial settings where conventional adhesives often fail.

### Mussel-inspired bioadhesives

Catechol-functionalized hydrogels are synthetic polymer networks inspired by the DOPA residues in Mfps, incorporating catechol groups that enable multiple adhesion mechanisms. These groups facilitate strong hydrogen bonding with polar surfaces, metal ion coordination (*e.g.*, Fe^3+^) to form reversible crosslinks, and covalent bonding through quinone formation upon oxidation (Fig. 4). Additionally, aromatic rings in catechols contribute via π–π and hydrophobic interactions, enhancing overall adhesion and mechanical stability. The mechanical properties of these hydrogels are highly tunable, as variations in polymer backbone, catechol density, and crosslinking methods allow control over stiffness, elasticity, and swelling behavior, while metal–catechol and covalent crosslinking improve cohesion and toughness under stress. Such features make catechol-functionalized hydrogels highly suitable for biomedical applications, including tissue adhesives, wound healing, drug delivery, and conformal coatings for medical devices, providing strong wet adhesion, biocompatibility, and structural integrity. For example, a chitosan–catechol bioadhesive polymer was developed by conjugating catechol onto chitosan, which dramatically enhanced its solubility [114]. This improved solubility allowed the catechol groups to mimic the adhesive behavior of mussel proteins more effectively. The resulting chitosan–catechol polymer was biocompatible and demonstrated excellent hemostatic properties as well as strong tissue adhesion, making it suitable for medical applications.

**Fig. 6:**
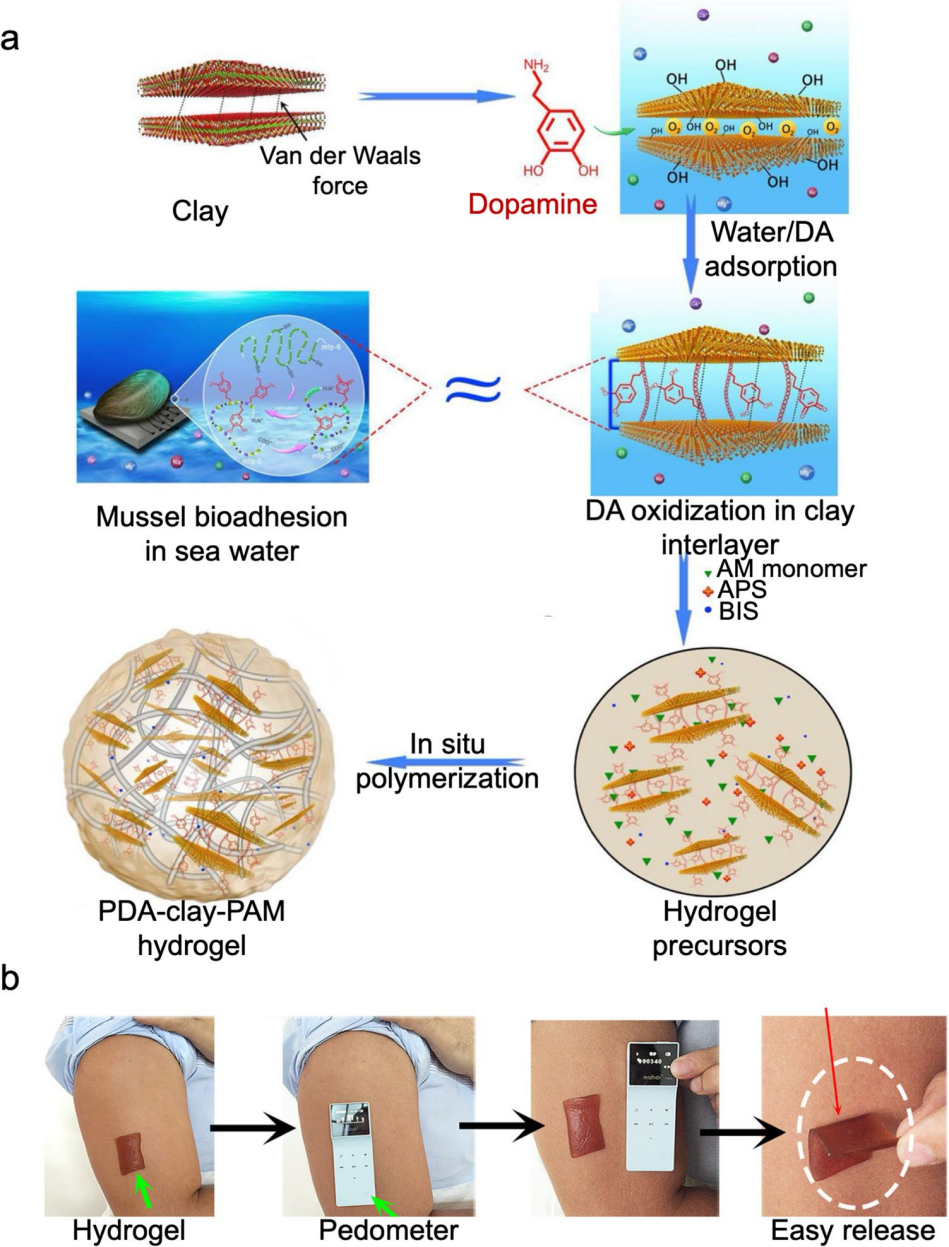
Mussel-inspired bioadhesive. **a** Design strategy for the preparation of PDA–clay–PAM hydrogel. **b** The hydrogel tape adhered directly to human skin and could be easily removed without causing irritation, allergic reactions, or leaving any residue. Reproduced with permission from [115]

Similarly, a polydopamine–clay–polyacrylamide (PDA–clay–PAM) hydrogel was developed via a two-step process [115]. Dopamine was intercalated into clay nanosheets and partially oxidized to retain free catechol groups, followed by in situ polymerization of acrylamide to form the hydrogel (Fig. 6). Unlike conventional single-use adhesives, this hydrogel exhibited durable, repeatable adhesion directly on human skin without inflammation or damage upon removal. The hydrogel’s adhesive properties were inspired by mussel byssal plaques, where densely packed catechol groups in nanoscale spaces drive strong attachment. The hydrogel also demonstrated enhanced toughness through clay nano-reinforcement and PDA-mediated network interactions, supported cell attachment and proliferation, and proved effective as a dressing in a rat full-thickness skin defect model. This free-standing, adhesive, tough, and biocompatible hydrogel offered practical advantages for surgical applications compared to in situ gelation adhesives.

Moreover, mussel-inspired materials exploit molecular interactions and include a variety of bulk adhesives, such as nanometer-thick polymer coatings, plastic films, elastomers, adhesive tapes [116, 117], hydrogels [118], and sensors [119]. Their adhesive performance depends on both material properties and bonding mechanisms, which involve physical interactions, such as hydrogen bonding, electrostatic forces, and host–guest interactions as well as chemical reactions including Michael additions, Schiff-base formation, and C–H insertion.

### Barnacle-inspired bioadhesives

Based on the adhesion principles and structural composition of barnacle cement, various barnacle-inspired adhesives have been developed, including recombinant CPs and peptides [93, 120, 121], and CP-mimetic bioadhesives [122, 123].

**Fig. 7:**
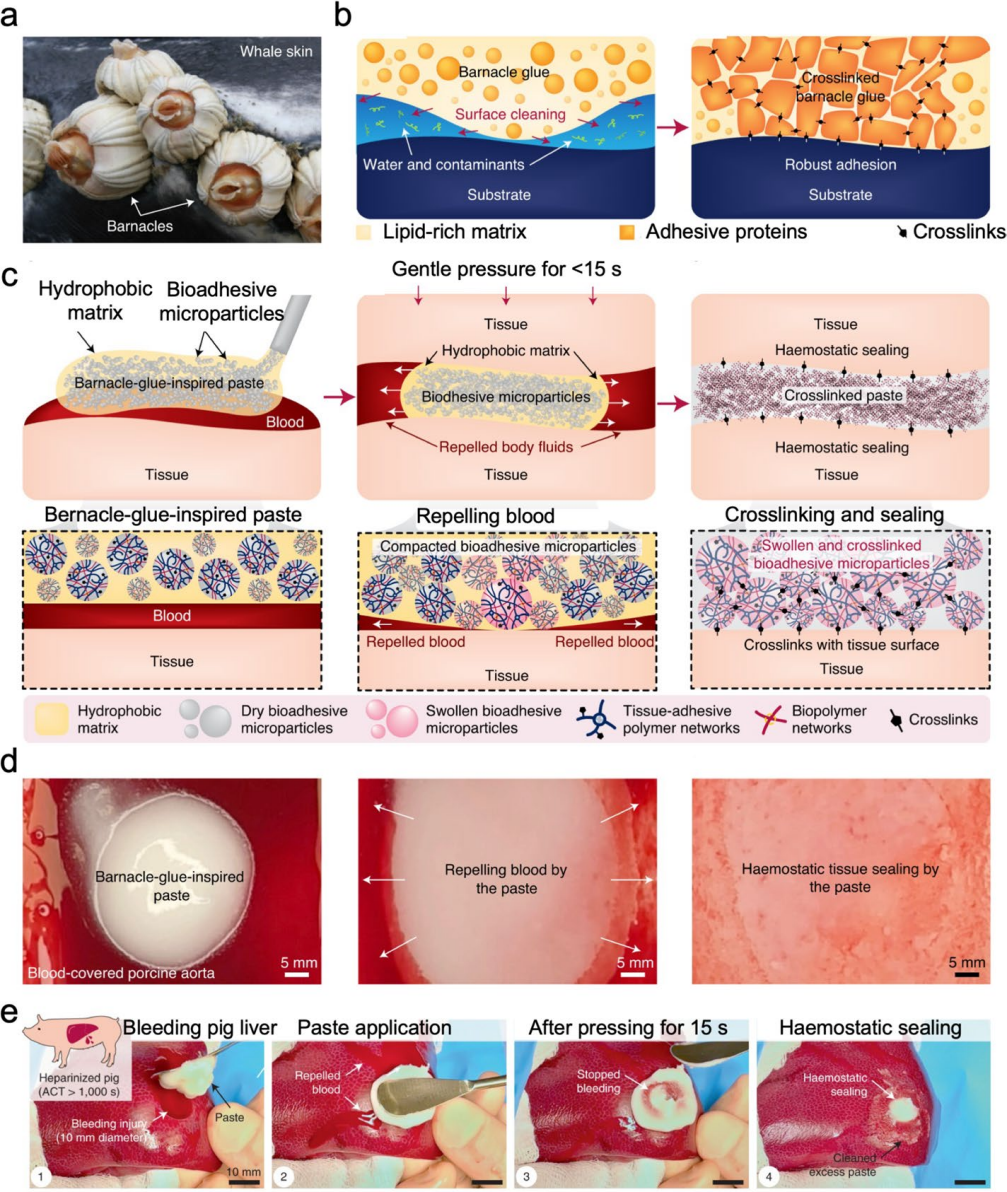
Barnacle-inspired bioadhesive. **a** Barnacles firmly attached onto the skin of a whale. **b** Schematics of barnacle glue, where a lipid-rich matrix which repels water and contaminants, enabling adhesive proteins to crosslink and form strong bonds on the cleaned substrate. **c** Schematics of a barnacle glue-inspired paste composed of bioadhesive microparticles within a hydrophobic oil matrix, with a repel–crosslinking mechanism that repels blood and forms hemostatic seals through protein crosslinking. **d** Photographs showing the barnacle-glue-inspired paste applied to a blood-covered porcine aorta, pressed with a gelatin-coated glass substrate, and forming a hemostatic tissue seal. **e** Rapid hemostatic sealing of a bleeding liver injury using the barnacle glue-inspired paste in a fully anticoagulated pig following systemic heparin administration. Reproduced with permission from [124]

In wet environments, the interfacial hydration layer represents a significant barrier to effective adhesion. Barnacles overcome this barrier by first releasing a hydrophobic lipid-rich matrix to remove water and contaminants, then depositing proteinaceous adhesives to form robust adhesion. Inspired by the spatiotemporal bioadhesion process of barnacles, an injectable hemostatic bioadhesive paste was developed [124]. It consisted of bioadhesive microparticles made from crosslinked poly(acrylic acid) grafted with *N*-hydroxysuccinimide ester (PAA-NHS) and chitosan, and hydrophobic oil matrix (Fig. 7). Upon application to bleeding tissues, the oil matrix repelled blood under mild pressure (10 kPa), while microparticles formed rapid hydrogen-bonded sealing. Over time, the NHS groups of PAA-NHS reacted with amine groups on the tissue surface and chitosan, leading to covalent crosslinks that provided stable and durable tissue sealing. The paste exhibited favorable biocompatibility and strong adhesion to various wet tissues. It outperformed commercial hemostatic products in controlling bleeding from porcine aortas ex vivo and in sealing bleeding heart and liver tissues in live rats and anticoagulated pigs, demonstrating the paste’s coagulation-independent hemostatic capability.

Similarly, inspired by the structural features of barnacle wet adhesion and utilizing solvent exchange, a robust barnacle CP-based wet adhesive hydrogels have been developed. CPs within bulk cement (*e.g.*, cp52k) are rich in cationic arginine and lysine, and aromatic phenylalanine and tyrosine, enhancing cohesion via hydrophobic and cation–π interactions, while interface CPs (*e.g.*, cp19k) contain cationic lysine and hydrophobic residues (valine, leucine, etc*.*) that strengthen electrostatic adhesion [56]. A bioadhesive hydrogel was developed using cationic 2-(acryloyloxy)ethyl trimethylammonium chloride (ATAC) and aromatic 2-phenoxyethyl acrylate (PEA) via simple free-radical copolymerization in dimethyl sulfoxide (DMSO), followed by swelling in water [125]. The resulting hydrogel exhibited high toughness through interchain π–π and cation–π interactions and strong wet adhesion via cooperative cation–aromatic interfacial interactions. It demonstrated strong wet adhesion, making it promising for underwater repair and wound dressing applications. Likewise, a chitosan–PEA hydrogel was synthesized, and its anti-swelling properties were achieved through solvent exchange between DMSO and water [123]. The chitosan–PEA hydrogel demonstrated strong wet adhesion to various underwater interfaces, adapted well to joint movements and skin twisting, and effectively sealed damaged organs.

## Bioinspired adhesive structural designs

Over the past few decades, a wide range of bioinspired structural adhesives have been developed to emulate the bioadhesive systems found in nature, such as tree frog toe pads, octopus suckers, and gecko toe pads, including many other specialized biological structures (Fig. 6). In this section, we highlight recent progress in biomimetic adhesive surface surfaces inspired by diverse natural bioadhesion mechanisms.

### Tree frog-inspired adhesive patterns

**Fig. 8:**
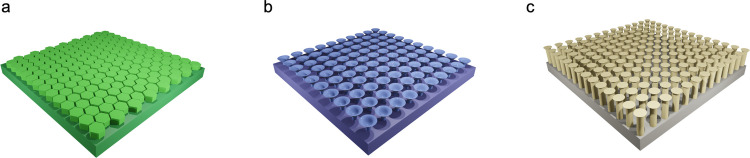
Bioinspired structural adhesives. **a** Polygon array inspired from tree frog toe pads. **b** Sucker array inspired from octopus’s suckers. **c** Seta array inspired from gecko setae

The polygonal micropillar arrays found on tree frog’s toe pads enable them to attach in humid environments (Fig. 8a). Along with van der Waals interactions from direct surface contact, adhesion is further enhanced by capillary forces generated through liquid channels between the micropillars and the substrate. The strength of these capillary forces is highly dependent on the thickness of the liquid film, which in turn is influenced by the arrangement of the micropillars. The capillary force is strongly influenced by the thickness of the liquid film, which is regulated by the geometric arrangement of the micropillars. This configuration controls liquid drainage and distribution, thereby determining the film’s morphology and uniformity. Accordingly, optimizing the design of tree frog-inspired micropillar arrays is crucial for enhancing adhesion under wet conditions. In fact, diverse micropatterns of polygonal micropillar arrays have been observed on toe pads across the species of tree frogs [97]. Current studies in biomimetic wet adhesives have therefore focused on tuning the shape, dimension, and hierarchical organization of micropatterns to replicate the bioadhesion of tree frog pads [26, 126].

In a study, seven surface patterns were systematically compared and hexagonal micropillars were found to provide the strongest adhesion due to superior channel drainage, which reduced liquid film thickness and enhanced friction [127]. It was further shown that elongated hexagons outperformed regular ones under wet conditions, as their lower bending stiffness and higher edge density improved friction [128]. Concave T-shaped tips were further tested and capillary forces were confirmed to dominate wet adhesion, with terminal structures having little effect [129]. Moreover, the dimensions of micro- and nanopillars also play a crucial role in determining wet adhesion. It was reported that pillars with heights of 5–10 μm revealed minimal differences in friction; however, when the height increased to 20 μm, excessive deformation occurred, markedly reducing friction under wet conditions. This deformation promoted pillar aggregation, which in turn decreased the effective contact area [128]. Importantly, adhesion was demonstrated not to be governed by a single parameter but by the combined influence of pillar height, width, and length ratios, which together dictate effective contact area and drainage capacity [130].

### Octopus-inspired adhesive suckers

The octopus is a well-known marine organism that uses suction cups for strong underwater adhesion. This adhesion is primarily driven by suction forces, which depend on the pressure differential and the effective contact area. Maximizing adhesion therefore requires secure sealing and adaptability to diverse contact surfaces. Efforts to replicate octopus’s sucker-like bioadhesion have emphasized tuning cavity design, particularly their curvature, form, scale, and hierarchical arrangement (Fig. 8b).

One report replicated the infundibular and circumferential rims of octopus suckers, producing bioinspired designs with excellent adaptability for underwater adhesion [131]. Their suckers had curved cavities to reduce edge modulus and improve sealing, increase contact area and optimal switchable adhesion. A small-scale soft robot with octopus-inspired adhesive property was developed by integrating poly(*N*-isopropylacrylamide) (PNIPAM) hydrogel into dome-like protuberances within a polyethylene glycol-diacrylate hydrogel sucker structures [132]. Mimicking the natural roof protuberance of octopus suckers, the microdomes generated capillary-assisted forces to enhance wet adhesion, while the temperature-responsive volume change of PNIPAM allowed reversible, on-demand detachment by adjusting the temperature. In atmospheric conditions, suction strength is limited by compressible air (10–40 kPa), but adhesion increases substantially in liquids due to incompressibility. To overcome this, two-photon lithography was used to print three-dimensional (3D) hybrid structures combining octopus- and gecko-inspired features, achieving strong, reversible adhesion on both wet and dry surfaces [133]. Regarding the size effects, it was found that adhesion scaled with sucker area, though large suckers suffered leakage from poor conformity [134]. In contrast, smaller suckers were observed to sustain higher pressure differentials, as reduced circumferential stress allows them to withstand greater loads [135].

Moreover, an octopus-inspired underwater adhesive system was developed by integrating switchable adhesive units with sensing, processing, and control components, allowing for fully autonomous attachment and release [136]. Each adhesive unit consisted of a silicone stalk and a pneumatically actuated membrane, allowing adhesion to be activated by negative pressure and deactivated by positive pressure. These adhesive units were coupled with micro-light detection and ranging optical proximity sensors and a microcontroller for real-time object detection and adhesion control, enabling intelligent modulation of multiple adhesive elements for precise manipulation in wet environments. The system achieved rapid, reversible switching (< 50 ms), underwater adhesive stresses exceeding 60 kPa, and an adhesion on/off ratio of over 450 ×. This functionality was demonstrated in a wearable adhesive glove, which successfully picked up and released a variety of underwater objects, including flat, curved, rigid, and soft objects.

### Gecko-inspired adhesives

**Fig. 9:**
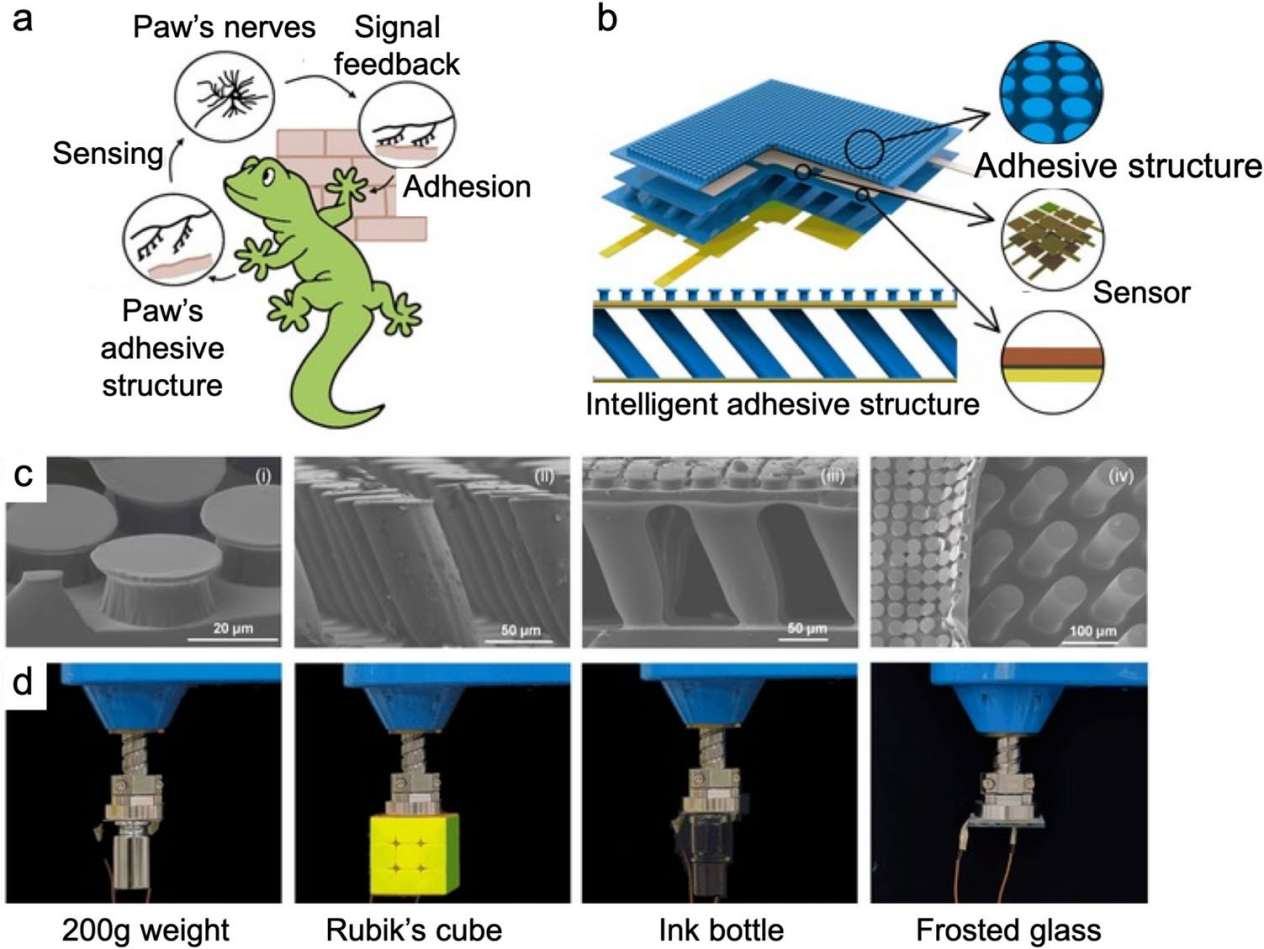
Gecko-inspired adhesive structure. **a** The crawling of a gecko is realized by joint action of the adhesion system of its toe pads and nervous system. **b** An integrated adhesion and sensing structure combines a mushroom-shaped layer with tilted micropillar supports, providing hierarchical bionic dry adhesion and enabling capacitive sensing via electrodes on both sides. **c** Scanning electron micrographs showing the integrated adhesion and sensing structures. **d** Demonstration of adhesion of bionic dry adhesive structures with smooth surfaces (200-g weight and Rubik’s cube), concave surfaces (the bottom of an ink bottle), and rough surfaces (frosted lass). Reproduced with permission from [138]

Geckos achieve strong adhesion through the hierarchical structure of their feet, where each tiny hair branches into multiple finer structures that maximize contact area with surfaces (Fig. 8c) [137]. In fact, gecko locomotion is achieved through the coordinated interaction between the adhesive structures on its toe pads and its nervous system [138]. The hierarchical setae and spatulae on each toe provide strong, reversible adhesion, while the nervous system precisely controls muscle movements to attach and detach individual toes. This integration allows the gecko to dynamically modulate adhesion, enabling efficient crawling, rapid climbing, and stable movement on vertical or inverted surfaces (Fig. 9a). Various gecko-inspired biomimetic structures have been created, replicating the multi-scale architecture of gecko toe pads [139, 140]. For example, a polydimethylsiloxane (PDMS)-based integrated adhesion and sensing structure was developed, featuring a mushroom-shaped layer supported by tilted micropillars and combining hierarchical bionic dry adhesion with capacitive sensing [138]. The structure consisted, from top to bottom, of a bionic adhesive layer, flexible electrodes, an inclined support layer, additional electrodes, and a PDMS substrate (Fig. 9b). The tilted micropillar support enhanced surface adaptability and enabled pressure-based sensing. Compared to vertical or unsupported supports, the inclined pillars exhibited superior compression, allowing the sensor to detect small deformations more effectively while maintaining stability and reliability in variable or extreme conditions (Fig. 9c). The hierarchical bionic dry adhesive structure adhered effectively to objects with varying surface characteristics (Fig. 9d). The structure showed excellent adaptability to smooth surfaces (*e.g.*, weights, Rubik’s Cube) and maintains strong adhesion on concave (*e.g.*, ink bottles) and rough surfaces (*e.g.*, frosted glass).

Similarly, an adhesive system was designed to investigate how incorporating soft backings into microfiber-reinforced adhesives enhances performance on curved surfaces by mimicking the micropatterned toe pads of tree frogs and the microfibrillar structures of gecko toes [141]. Polyurethane foams were used as soft backings to improve the conformability of the adhesive on curved surfaces, while silicone hydroskeletons provided an additional soft and viscoelastic layer. These soft backings were reinforced with polyester mesh for structural support, and a smooth silicone layer was incorporated to facilitate mounting and ensure uniform contact with the substrate.

Another recent study introduced a digital light processing (DLP) printing process that used resin overcuring to generate 3D gecko seta-inspired anisotropic pillar structures [142]. The method reduced hierarchical complexity and produced smooth, stable surfaces for directional adhesion and detachment. Using a double-casting technique, these structures achieved gecko-like adhesive strength with easy release, and a simple mechanical module demonstrated the detachment effect. This approach offered a novel, compatible strategy for fabricating enhanced anisotropic surfaces with existing 3D printing technologies.

## Applications of bioadhesives in regenerative medicine

Natural bioadhesives derived from organisms, such as mussels and salamanders, offer unique advantages for tissue engineering and regenerative medicine due to their biocompatibility, strong wet adhesion, and tunable mechanical properties [143, 144]. These adhesives can securely bond tissues without the need for sutures or staples, providing minimally invasive solutions for wound closure, hemostasis, and organ repair. In addition, their ability to form conformal, reversible, or degradable bonds makes them highly suitable for supporting cell attachment, proliferation, and tissue integration in engineered scaffolds. By mimicking the chemical and physical mechanisms of natural adhesion, researchers have developed bioinspired hydrogels, films, and sealants that promote tissue regeneration while reducing inflammation and tissue damage, expanding the potential of natural adhesives in clinical applications.

Millions of traumatic and surgical wounds require prompt closure each year [145]. Traditional sutures and staples often cause secondary injury and scarring, prompting growing use of sutureless methods [146]. Non-toxic and biocompatible adhesives provide rapid tissue binding, reduce pain, and minimize scar formation. Recently, various mussel-inspired functional hydrogels have been widely used to promote wound healing [147–150]. For example, a hydrogel was developed, in which catechol-modified *ε*-poly-l-lysine (PL-Cat) and oxidized dextran (ODex) acted as natural polymer backbones, crosslinked in situ through Schiff base and catechol–Fe coordination bonds [151]. This dual dynamic bonding imparted strong adhesion, mechanical strength, rapid dissociation, and self-healing properties. The hydrogel offered dissolution on demand, repeatable adhesion, injectability, and biocompatibility. It effectively closed skin incisions, could be easily removed, and enabled repeated wound closure for post-care management. Similarly, a bioglass/oxidized sodium alginate hydrogel was produced that enhanced vascularization and tissue regeneration [152]. Bioglass strengthened tissue bonding by creating an alkaline environment that promoted oxidized sodium alginate-tissue interactions, while the released Ca^2+^ enabled adhesion to implantable materials through chelation with hydrogel carboxyl groups. Moreover, mussel-inspired antimicrobial hydrogels were designed using catechol-functionalized oxidized hyaluronic acid, guar gum, glycol chitosan, borax, and polydopamine nanoparticles (PDA NPs) [153]. Along with strong adhesion, injectability, and self-healing, the incorporation of PDA NPs endowed the hydrogels with near infrared (808 nm)-activated photothermal antimicrobial activity, making them effective for healing bacteria-infected wounds. In another example, multifunctional hydrogel dressings with antibacterial and antioxidant properties were created by combining chitosan functionalized with polyethylene glycol monomethyl ether-glycidyl methacrylate, methacrylamide dopamine, and zinc ions [154]. These hydrogels demonstrated strong hemostatic performance in mouse liver hemorrhage and tail amputation models, while also exhibiting antibacterial activity against Methicillin-resistant *Staphylococcus aureus*. Their tissue-adhesive properties and effective volume contraction facilitated rapid wound closure and accelerated healing, highlighting their potential as advanced dressings for infected or challenging wounds.

Beyond wound healing and hemostasis, drug delivery is a key function of emerging bioadhesives. Bioadhesive intraperitoneal (IP) patches were developed by synthesizing catechol-grafted chitosan conjugates via the 1-ethyl-3-(3-dimethylaminopropyl)carbodiimide hydrochloride coupling reaction [155]. The partially crosslinked IP (c-IP) patches were prepared by incubating the catechol–chitosan solution at 40 °C for 12 h. The IP patch adhered strongly to wet peritoneal tissue (~ 42 kPa), while the c-IP patch showed slightly lower adhesion (~ 34 kPa). Both patches were loaded with 5-fluorouracil and applied in a mouse peritoneal cancer model. The c-IP patch, with its partially crosslinked network enabling sustained drug release, demonstrated superior anticancer efficacy, highlighting the potential of such multifunctional adhesive patches as drug carriers for cancer therapy.

While bulk scaffolds have been widely used as biomaterial grafts for large bone defects, they are often inadequate for fragmented or comminuted fractures [156]. Injectable bioadhesives offer a practical alternative, enabling the filling and stabilization of such complex defects [157]. In addition, bioadhesives can serve as a glue to secure autologous or bioengineered bone grafts to host tissue [158]. Conventional fixation methods, including screws, wires, or plates, are generally unsuitable for powdered or small grafts in comminuted fractures. Numerous bioadhesives have shown promising biocompatibility, biodegradability, and mechanical strength, making them a versatile solution for enhancing bone graft fixation and promoting effective bone healing.

In addition to mussel-inspired hydrogel adhesives for drug delivery, biomimetic adhesives inspired by biological surface structures have also been reported. For example, a glucose-responsive microneedle (MN) patch was designed for insulin delivery, incorporating red blood cell vesicles or liposomes with glucose transporters (GLUTs) bound to glucosamine-modified insulin (Glu-Insulin) [159]. The MNs could penetrate the skin, releasing insulin in response to elevated glucose levels through competitive binding with GLUTs. Supplementing with free Glu-Insulin ensured sustained release. In diabetic mice, this smart GLUT-based patch effectively maintained long-term blood glucose control. Similarly, inspired by the hunting strategy of the blue-ringed octopus, a wet-adhesive MN patch was developed for controlled drug delivery [160]. Pluronic-F127 (PF127)-based hydrogel suction cups were integrated onto silk fibroin to generate silk fibroin-PF127 (Silk-Fp) patch, which would provide wet adhesion, with the inner surfaces treated with tannic acid to enable biocompatible chemical bonding. These Silk-Fp MNs allowed precise drug delivery, carrying anti-inflammatory agents like dexamethasone for oral ulcers or anticancer drugs such as 5-fluorouracil for early-stage tumors. This biomimetic patch demonstrated stable wet adhesion and effective intratissue drug administration, supporting accelerated wound healing or tumor inhibition.

In addition, the mussel-inspired bioadhesion mechanism was used to develop bone adhesives for xenograft bone substitutes [158]. DOPA-containing mussel adhesive proteins (MAPs) were formulated that effectively maintained adhesion of deproteinized bovine bone mineral (DBBM) particles. Compared to controls, including tissue culture plates, MAP without DOPA, and poly-L-lactide, the DOPA-MAP adhesive enhanced osteogenic differentiation of MC3T3-E1 osteoblasts. Implantation of DBBM aggregates bound with DOPA-MAP into critical-sized rat calvarial defects significantly promoted bone formation within 8 weeks, outperforming DBBM alone or untreated controls.

Recently, a catechol-based dual-network nerve adhesive (DNNA) was developed by conjugating dopamine–isothiocyanate, which contained both catechol and thiourea groups, to hyaluronic acid [161]. The resulting conjugate was then combined with a decellularized peripheral nerve matrix, and enzymatic gelation was achieved using mushroom tyrosinase through quinone-thiourea couplings. Unlike quinone-–quinone interactions, these couplings are more efficient and robust, while also reducing quinones back to catechols. This mechanism enabled DNNA to gel rapidly, achieve strong adhesion, and minimize quinone accumulation. The biocompatibility of DNNA was confirmed in vitro through Schwann cell proliferation and dorsal root ganglion neurite outgrowth assays. The therapeutic efficacy of DNNA was evaluated in a rat sciatic nerve transection model. At 10 weeks post-surgery, the strong adhesion and bioactivity of DNNA significantly reduced intraneural inflammation and fibrosis, enhanced axonal reconnection and remyelination, promoted motor and sensory function recovery as well as improved muscle contraction, compared to conventional suture and fibrin glue.

Mussel-inspired adhesives have also been explored for their potential applications in adhesive dentistry [162, 163]. In a study, a PDA-based zinc (Zn)-containing dental adhesive was synthesized to coat silicon dioxide (SiO_2_) particles [164]. The resulting Zn–PDA–SiO_2_ particles were used as dental adhesive, which exhibited antibacterial properties as well as inhibition of bioenzymatic activity from both soluble and matrix-bound proteases. The Zn ions and catechol groups within the Zn–PDA–SiO_2_ structure conferred enhanced antibacterial and antienzymatic activities, leading to long-lasting dentin bonding efficacy. Similarly, *N*-(3,4-dihydroxyphenethyl) methacrylamide (DMA) has been shown to preserve dentin bond strength when applied to etched dentin surfaces [165]. In addition, DMA primers have demonstrated the ability to enhance dentin shear bond strength and reduce microleakage [166]. Moreover, the application of a poly(dopamine-methacrylate-co-2-methoxyethyl acrylate) primer improved bonding to saliva-contaminated dentin, and the incorporation of Fe^3+^ further elevated bond strength to levels considered clinically acceptable [167]. In a recent study, the catechol–Lys–methacrylate (CLM) primer was effectively grafted onto caries-affected dentin and was shown to increase immediate bond strength while reducing leakage [168]. CLM chemically modified the collagen matrix, promoting collagen crosslinking, inhibiting endogenous enzymatic activity, and imparting antibacterial properties, thereby further enhancing the stability of the bonding interface.

Likewise, a dual-bionic hydrogel inspired by mussels and barnacles was designed by combining catechol-conjugated chitosan, tannic acid, and silk fibroin (C-CTS) [169]. To further prevent wound infection and accelerate tissue healing process, sodium alginate (SA)-coated silver (Ag) nanoparticles (SA-Ag) and liver decellularized extracellular matrix (dECM) were introduced into the C-CTS hydrogel, resulting into C-CTS/SA-Ag/dECM hydrogel. The C-CTS/SA-Ag/dECM hydrogel showed ultrahigh adhesion performance on various wet substrates due to hydrogen bonding, electrostatic force, and cation–π interactions. Besides sufficient mechanical strength and repeatable adhesion, antibacterial and self-healing properties were also observed. C-CTS/SA-Ag/dECM hydrogel was shown better hemostatic capability in a variety of arterial hemorrhage rabbit and porcine models compared with the commercial gauze due to the synergistic effect of robust wound sealing, efficient red blood cell capture, and activation of the hemostatic barrier.

Thus, biomimetic and nature-inspired adhesives represent a promising frontier in tissue repair and regenerative medicine. Significant advancements in wound healing can be achieved, including the reduction or even prevention of scar formation. These bioadhesives, which mimic the properties of natural bioadhesives such as mussel proteins, offer multiple therapeutic benefits. Not only do they enhance tissue regeneration by supporting cellular adhesion and proliferation, but they also exhibit inherent anti-inflammatory and antibacterial properties, reducing the risk of infection and inflammation at the wound site. Moreover, their ability to maintain a moist and protective microenvironment is critical for optimal healing, as it facilitates cell migration and prevents desiccation. In addition to these wound-healing functions, many of these bioadhesives can serve as effective drug delivery platforms, allowing for the localized and sustained release of therapeutic agents directly at the wound or tumor sites.

Altogether, natural and nature-inspired adhesives derived from both aquatic and terrestrial animals exhibit remarkable adhesion strategies that have inspired the development of next-generation bioadhesives with a wide range of potential applications, including wet-tissue bonding, hemostasis, wound healing and tissue repair, underwater repair, and device integration, among others. Despite their promise, these systems possess certain limitations that hinder their direct translation to practical biomedical applications (Table 1). Bioinspired adhesives generally face trade-offs between adhesion strength, repeatability, and environmental robustness. For example, mussel- and barnacle-inspired adhesives provide strong wet adhesion but are often limited by low load-bearing capacity, slow curing, and challenging in replicating complex protein structures. Tree frog- and octopus-inspired systems are effective on wet surfaces but may lack mechanical strength and durability under repeated use. Gecko-inspired adhesives achieve remarkable dry adhesion but often perform poorly in wet conditions, while salamander-inspired adhesives are temporary and low in load-bearing. Furthermore, integrating multifunctional properties such as self-healing, antibacterial activity, and biocompatibility remains challenging, and scalable biofabrication of hierarchical structures is difficult. These limitations highlight the need for hybrid strategies that combine the strengths of multiple natural bioadhesion systems to achieve versatile, high-performance bioadhesives suitable for biomedical applications.

**Table 1:** Comparative overview of main bioadhesive strategies and their applications

Organisms	Adhesive molecules/structures	Bioadhesion mechanisms	Adhesion type	Environmental adaptations	Exemplary applications	Limitations	References
Mussel	DOPA (catechol)-rich Mfps	Wet adhesion via hydrogen bonding, metal coordination, and covalent bonding	Permanent	Strong adhesion in marine environment	Surgical wet-tissue glues, dental adhesives, sensors, drug delivery patches	Requires controlled catechol oxidation, involves complex proteins, and slow curing or limited long-term reliability	[3, 57, 75–77, 79–83, 115, 117, 119, 158, 159, 162–168]
Barnacle	Phosphorylated CPs and lipids	Wet adhesion via electrostatic and disulfide bonding	Permanent	Strong adhesion in turbulent marine environment	Medical sealants, antifouling coatings	Hard to remove, complex multi-protein system, challenging to synthetically replicate CP sequence and structure	[56, 85–87]
Tree frog	Glycoproteins, mucopolysaccharides in mucus and toe pad microstructures	Toe pad microstructures and mucus-mediated adhesion via capillary and hydrodynamic forces	Temporary/reversible	Effective adhesion on both dry and wet surfaces	Wet-surface grip pads, prosthetic adhesives	Toe pad patterns hard to replicate, prone to mucus drying and contamination	[29, 63]
Octopus	Proteins, glycoproteins in mucus, and muscular suction cups	Muscular suction and mucus-mediated wet adhesion through negative pressure and increased contact area	Temporary/reversible	Effective adhesion on rough, irregular and wet surfaces	Reusable underwater adhesives, soft robotic grippers, medical adhesive patches, surgical robots	Needs active actuation, suction ineffective on highly porous and rough surfaces	[21, 98, 100, 101]
Gecko	Microfibrillar setae with spatulae	Physical dry adhesion via van der Waals forces	Temporary/reversible	Specialized for adhesion on dry, rough and vertical surfaces	Reusable surgical tapes, robotics microgrippers	Adhesion reduces on wet surfaces, requires close conformal contact, large-scale synthetic setae fabrication challenging	[138–140, 142]
Salamander	Glycoproteins and peptides in skin secretions	Mucus-mediated adhesion via capillary forces and viscous interactions	Temporary/reversible	Effective adhesion on diverse dry substrates and vertical surfaces	Biocompatible glues, surgical dressings	Adhesion reduces on wet surfaces, requires intimate substrate contact	[30, 32, 84, 104, 105]

*Abbreviations*: *CPs* cement proteins, *DOPA* 3,4-dihydroxyphenylalanine, *Mfps* mussel foot proteins

## Conclusions and future perspectives

The field of bioadhesives has progressed rapidly in recent years, driven by advances in mechanics, biomaterials science, and biology. These developments have given rise to bioadhesives for regenerative medicine that move beyond conventional mechanical support to actively engage biological processes and promote tissue healing and regeneration. By tuning their mechanical properties to specific tissues and incorporating biological cues that regulate cell behavior, these systems open broad design possibilities for balancing strength, flexibility, and bioactivity to achieve more comprehensive tissue regeneration.

Most commercial bioadhesives still function primarily as passive mechanical sealants, but there is growing potential to engineer formulations that directly facilitate repair. Because their mechanical properties can shift from static to dynamic, even subtle differences may yield significant biological outcomes. The challenge lies in optimizing these features, as the process is costly, time-consuming, and further complicated by the diverse biological environments bioadhesives encounter, including extracellular matrix remodeling and immune modulation. As a result, regenerative bioadhesives remain in an early stage of development, requiring systematic validation of their physical, chemical, and biological interactions.

Future research on bioadhesives should account for the diverse physiological contexts in which they are applied, including tissue type, scale, and regenerative functions. Biomaterial innovations will be central for advancing this field, and in line with this, new candidates such as dECM, donor-derived scaffolds, bacteria-based living systems, and DNA hydrogels are emerging. Among these, decellularized materials are especially attractive because they precisely replicate the biochemical characteristics of native tissues and provide tunable functional groups that can be customized for enhanced bioadhesion.

Additive manufacturing technologies, particularly 3D printing and bioprinting, also offer powerful tools to create bioadhesives with precise shapes, architectures, and functions. Bioprinting enables the integration of cells and bioactive molecules, yielding constructs that more closely mimic native tissues. Yet challenges persist in printing fragile living cells and biomolecules, adapting to irregular wound geometries, and ensuring long-term stability, mechanical integrity, and compatibility. Addressing these issues will be essential for clinical translation.

Another key direction is improving delivery methods. Injectable hydrogels, catheter-compatible formulations, and adhesive patches provide avenues for minimally invasive application. Success in this area depends on designing systems that function under real surgical conditions, such as saline-rich environments, and may require new applicators analogous to suturing tools.

Equally important is the capacity to modulate adhesive and mechanical behaviors on demand. Stimuli-responsive systems, triggered by temperature, light, ultrasound, or chemical signals, are being explored, though their long-term reliability in vivo remains uncertain. Developing robust response mechanisms will likely require novel chemical strategies.

In conclusion, bioadhesives for tissue regeneration represent a transformative opportunity for tissue repair and regenerative medicine. By integrating mechanical design with biological functionality, expanding material choices, advancing manufacturing and delivery approaches, and addressing regulatory pathways, these systems have the potential to reshape clinical practice and significantly improve patient outcomes.

## Data Availability

This manuscript has no associated data.
